# Fatty acids in breast milk during lactation in *Ateles geoffroyi* offspring in managed care and semi-free-ranging

**DOI:** 10.1093/cz/zoaf061

**Published:** 2025-09-08

**Authors:** Ma de Jesús Rovirosa-Hernández, María Remedios Mendoza-López, Manuel Alejandro Cruz Aguilar, Juan Francisco Rodríguez-Landa, Mario Caba, Rael M Palestino-Sánchez, Francisco García-Orduña

**Affiliations:** Instituto de Neuroetología, Universidad Veracruzana, Avenida Dr. Luis Castelazo, Industrial de las Ánimas, Rubí Ánimas, Xalapa CP 91190, México; Instituto de Química Aplicada (IQA), Universidad Veracruzana, Avenida Dr. Luis Castelazo, Industrial de las Ánimas, Rubí Ánimas, Xalapa CP 91190, México; Dirección de Investigaciones en Neurociencias, Instituto Nacional de Psiquiatría “Ramón de la Fuente Muñiz”, Calzada México-Xochimilco 101, Colonia, Huipulco, Tlalpan, CDMX CP 14370, México; Instituto de Neuroetología, Universidad Veracruzana, Avenida Dr. Luis Castelazo, Industrial de las Ánimas, Rubí Ánimas, Xalapa CP 91190, México; Sistema Nacional de Investigadoras e Investigadores (SECIHTI), Av. Insurgentes Sur 1582, Col. Crédito Constructor, Demarcación Territorial Benito Juárez, México CP 03949, México; Instituto de Neuroetología, Universidad Veracruzana, Avenida Dr. Luis Castelazo, Industrial de las Ánimas, Rubí Ánimas, Xalapa CP 91190, México; Instituto de Neuroetología, Universidad Veracruzana, Avenida Dr. Luis Castelazo, Industrial de las Ánimas, Rubí Ánimas, Xalapa CP 91190, México

**Keywords:** long-chain polyunsaturated fatty acids, maternal diet, spider monkeys, weaning behavior

Long-chain polyunsaturated fatty acids (LC-PUFAs) are precursors of omega-3 and omega-6, which are considered essential fatty acids (EFA), for pre- and post-natal neural development of several species including non-human primates (NHPs), (Hornstra 2000; Milligan and Bazinet 2008). The deficiency of these EFA can provoke growth retardation (Burr and Burr 1929), behavioral changes (Reisbick et al. 1994) and motor skill (Champoux et al. 2002). These LC-PUFAs coming from the mother diet (Huang and Brenna 2001), which have been reported in folivorous and omnivorous NHPs in manage care (Milligan and Bazinet 2008; Milligan et al. 2008a, 2008b). However, there is a lack of information on the milk fatty acids content in mainly frugivorous species, such as spider monkeys. The present report contributes to the knowledge of the influence of the frugivorous diet on the fatty acid content of breast milk and the weaning behavior of infants.

The milk of two lactating mothers (LM) was analyzed: LM1 was housed in wire-cloth cages (3 m length × 4 m height × 3 m width), and LM2 in an enclosure of 100 m diameters, containing secondary vegetation. Both mothers received daily diet of seasonal fruits and vegetables (chard, beets, apple, lemon, cucumber, carrot, banana, avocado, papaya, tomato, celery, mango, pineapple, orange). Additionally, LM2 has some trees that usually monkeys consume such as *Bursera simaruba, Ficus cotinifolia, Ficus lundelli, Ficus perforata,* and *Spondias mombin*.

Milk samples were obtained at 2 months of lactation (Milligan et al. 2008b). Each female was tranquilized with tiletamine-zolazepam 10 mg/kg IM (Zoletil 100®, Virbac Lab. Zapopan 45010, Mexico). Subsequently, they received oxytocin 0.65UI/Kg IM (Syntocinon®, Novartis Laboratories, Sandoz, SA de CV, Mexico) 15 min prior to milking (Milligan et al. 2008a). Nipples were cleaned and both mammary glands were gently pressed for milk ejection. About 1.5 mL from each breast in a sterile microtube and then were frozen at −20 °C until analysis.

Milk samples were analyzed through gas chromatography coupled with mass spectrometry (Hewlett Packard®, G1800B; Agilent Technologies, Mexico), (see supplementary methodological information; Egan et al. 1981).

Each mother's infants (ILM1 and ILM2) were recorded by the focal-animal method (Altmann 1974), during the first 8 months after birth (8:00 AM to 7:00 PM), both the time and frequency of milk consumption, as well as solid food, to identify the time of milk consumption and the beginning of solid food consumption (weaning).

Analysis of LM2 breast milk, showed a higher concentration of the fatty acids saturated (SFA), monounsaturated (MUFAs), and polyunsaturated (PUFAs), with respect to the milk of LM1 (Table 1). Also, the ILM2 infant showed 100% in the frequency and time of milk ingestion during the first 3 months after birth. In contrast, during the 4th month consumed 100% of wild food. In months 5 to 8, it showed a higher frequency and time in the consumption of solid food, particularly wild food, complementing with the intake of milk.

**Table 1.** Average percentage of fatty acids in the milk of *Ateles geoffroyi* and other reported primate species that are found in managed care

Fatty acid SFAs	*Ateles geoffroyi*			Callithrix jacchus (*n* = 4) (Milligan and Bazinet 2008)	Leontopithecus rosalia (*n* = 1) (Milligan and Bazinet 2008)	Cebus apella (*n* = 7) (Milligan and Bazinet 2008)	Saimiri bolivienses (*n* = 8) (Milligan and Bazinet 2008)	Macaca mulatta (*n* = 21) (Milligan and Bazinet 2008)	Saimiri boliviensis boliviensis (*n* = 3) (Milligan, et al. 2008b)
	LM1	LM2	(*n* = 2)						
C6:0	0.19	0.11	0.150 ± 0.056	ND	ND	ND	ND	ND	ND
C8:0	3.17	1.65	1.977 ± 1.068	ND	ND	ND	ND	ND	5.38 ± 0.30
C10:0	13.90	11.20	12.223 ± 1.464	ND	ND	ND	ND	ND	3.80 ± 0.29
C12:0	15.47	13.45	14.007 ± 1.279	ND	ND	ND	ND	ND	0.01 ± 0.01
C14:0	12.20	11.60	10.647 ±2.191	ND	ND	ND	ND	ND	2.60 ± 0.21
C16:0	18.22	21.79	20.013 ± 1.785	ND	ND	ND	ND	ND	17.54 ± 0.26
C18:0	5.42	5.32	5.370 ± 0.070	ND	ND	ND	ND	ND	4.31 ± 0.18
C20:0	ND	ND	ND	ND	ND	ND	ND	ND	0.11 ± 0.101
C22:0	ND	ND	ND	ND	ND	ND	ND	ND	ND
**Sum of saturates**	68.60	65.15	62.567 ± 7.659	ND	ND	ND	ND	ND	NR
C14:1	ND	ND	ND	ND	ND	ND	ND	ND	0.13 ± 0.004
C16:1	6.41	7.34	6.073 ± 1.464	ND	ND	ND	ND	ND	2.29 ± 0.16
C18:1	21.10	22.89	25.603 ± 6.314	ND	ND	ND	ND	ND	29.47 ± 0.57
C20:1	0.014	0.35	0.182 ± 0.238	ND	ND	ND	ND	ND	0.04 ± 0.01
C20:2	ND	ND	ND	ND	ND	ND	ND	ND	0.34 ± 0.01
C22:2	ND	ND	ND	ND	ND	ND	ND	ND	0.05 ± 0.01
**Sum of MUFAs**	27.53	30.59	31.803 ± 4.992	ND	ND	ND	ND	ND	NR
C18:3*n*−3	ND	ND	ND	2.21 ± 0.28	0.61	2.91 ± 0.14	1.65 ± 0.21	1.55 ± 0.11	2.08 ± 0.07
C20:5*n*−3	ND	ND	ND	0.04 ± 0.003	0.03	0.06 ± 0.004	0.09 ± 0.01	0.16 ± 0.01	ND
C22:5*n*−3	ND	ND	ND	0.19 ± 0.01	0.16	0.28 ± 0.02	0.18 ± 0.01	0.25 ± 0.01	0.22 ± 0.01
C22:6*n*−3	ND	ND	ND	0.15 ± 0.002	0.12	0.31 ± 0.04	0.40 ± 0.03	0.44 ± 0.02	0.45 ± 0.02
Sum *n*−3s	ND	ND	ND	2.59 ± 0.27	3.17	3.50 ± 0.09	2.32 ± 0.23	2.40 ± 0.52	ND
C18:2*n*−6	2.24	2.67	4.253 ± 3.122	21.67 ± 1.70	11.50	30.63 ± 1.35	30.39 ± 1.02	23.88 ± 0.54	28.78 ± 0.48
C18:3*n*−6	1.57	1.61	1.318 ± 0.472	1.20 ± 0.16	ND	0.10 ± 0.02	0.17 ± 0.02	0.09 ± 0.01	0.19 ± 0.01
C20:3*n*−6	ND	ND	ND	1.23 ± 0.11	0.86	0.37 ± 0.02	0.27 ± 0.01	0.25 ± 0.01	0.30 ± 0.02
C20:4*n*−6	ND	ND	ND	0.49 ± 0.02	0.36	0.92 ± 0.08	0.81 ± 0.04	0.44 ± 0.01	0.89 ± 0.03
C20:5*n*−3	ND	ND	ND	ND	ND	ND	ND	ND	0.11 ± 0.01
C22:4*n*−6	ND	ND	ND	ND	ND	ND	ND	ND	0.03 ± 0.01
C22:5*n*−6	ND	ND	ND	0.08 ± 0.002	0.10	0.10 ± 0.02	0.12 ± 0.01	0.07 ± 0.002	0.13 ± 0.01
**Sum *n*−6s**	3.85	4.25	5.571 ± 3.594	24.76 ± 1.72	25.88	32.01 ± 0.46	31.79 ± 1.00	24.76 ± 0.55	NR
**Sum of PUFAs**	**—**	**—**	5.574 ± 2.648	27.35 ± 1.96	29.04	35.61 ± 0.54	34.11 ± 0.89	27.15 ± 0.62	NR
**Total fatty acid (mg/g)**	**—**	**—**	6.755 ± 2.448	60.65 ± 11.04	15.90	59.12 ± 7.07	53.63 ± 6.33	74.94 ± 9.67	NR

ND, Non detected.

NR, Non reported.

While the ILM1 infant dedicated 100% in frequency and time of milk intake during the first 5 months after birth, in months 6 and 7, showed a higher frequency and time in the consumption of solid food, surprisingly in the month 8 the percentage of frequency and time was greater than 90, in the consumption of milk (Table 2).

**Table 2.** Monthly percentage of frequency and time of milk ingestion since birth of the ILM1 and ILM2 offspring, as well as the beginning of these by consumption of solid foods (wild and/or cultivated)

Offspring age after birth	% Frequency ILM1			% Time ILM1			% Frequency ILM2			% Time ILM2		
Months	Milk ingest	Solid food		Milk ingest	Solid food		Milk ingest	Solid food		Milk ingest	Solid food	
		W	C		W	C		W	C		W	C
1	100	—	—	100	—	—	100	—	—	100	—	—
2	100	—	—	100	—	—	100	—	—	100	—	—
3	100	—	—	100	—	—	100	—	—	100	—	—
4	100	—	—	100	—	—	—	100	—	—	100	—
5	100	—	—	100	—	—	60	40	—	22	78	—
6	60	—	40	52	—	48	29	71	—	23	77	—
7	75	—	25	30	—	70	45	45	10	14	72	14
8	97	—	3	96	—	4	58	23	19	21	51	28

C, Cultivated; W, Wild.

Breast milk has a rich nutritional content including protein components such as casein and whey proteins, immunoglobulins, and enzymes that contribute to the formation of the bifidus factor; non-protein components including taurine and nucleotides; immunomodulators, carbohydrates like lactose; energy supply, minerals and vitamins such as calcium, magnesium, iron, zinc, D, A, E, K, among others; cell maintenance and protection, lipids like oleic, palmitic, linoleic acids; important source of energy and development of the nervous system (Macías et al. 2006). However, the composition of milk and the duration of lactation in different mammalian species are adapted to the particular needs of each newborn (Muñoz 2001).

Regarding lipids in primates, we found the same fatty acids reported for other New World monkeys in wild and in managed care, (Milligan and Bazinet 2008; Milligan et al. 2008a, 2008b) with a notable exception the milk of spider monkeys mothers contain caproic acid 6:0, not reported for other New World primate species in managed care, but has been detected in Old World primates (Osthoff et al. 2009).

The SFAs (6:0 to 22:0), are synthesized by the mammary gland and are not reflective of supply in the maternal diet; however, it has been reported for NHPs, and other hominids, that diets high in fiber may be the basis for the conversion of SFAs to MUFAs (Popovich et al. 1997). *Ateles geoffroyi* is a highly frugivorous species, it has been suggested that the body can synthesize fatty acids from carbohydrates (German and Dillard 2010). A high carbohydrate intake promotes de novo lipogenesis of the PUFAs (Gómez-Cortés et al. 2018). It is suggested that SFAs in the milk of spider monkey mothers, could have been obtained from a diet rich in carbohydrates.

The MUFAs (14:1 to 22:2) are involved in many biological processes, such as maintaining memory and the optimal fluidity of the bilayer cell (Spector 1999), which are important during development. The milk of both mothers presents three MUFAs.

The body cannot synthesize the PUFAs (18:3*n*−3 to 22:5*n*−6); they are acquired in the diet that the mother ingests, and passed on to the fetus through the placenta and breast milk (German and Dillard 2010). These PUFAs are found in seed oils, and antioxidants such as flavonoids (Toufektsian et al. 2011). They are fatty acids critically necessary for the normal growth and development of the fetus until infancy, particularly for brain development and visual acuity (Neuringer et al. 1992, 1996; Luukkainen et al. 1994). It is suggested that the percentage of PUFAs that LM2 presented could have been obtained from the linoleic acid (C18:2 *n*−6, AL) and α-linolenic acid (C18:3 *n*−6, ALA) contained in the fruits of *Bursera simaruba* (Ferreira et al. 2019), *Ficus* sp (Knothe, et al. 2019), *Spondia mombin* (López-Camacho, et al. 2021), that mother consumed during her gestation and lactation. These fatty acids (AL and ALA) were detected in the milk of both mothers, with a concentration lower than that reported for New World primates, both in managed care and free-ranging populations (Milligan and Bazinet 2008; Milligan et al. 2008a). It is necessary to analyze the milk of females in the wild to determine whether these differences are also present in their natural state.

Weaning is the gradual transition period from the start of a solid diet to the withdrawal of the breast milk intake (Magne et al. 2006; Koenig et al. 2011). A study using stable isotopes in feces reports that the consumption of solid foods begins approximately at 2 months of age in the offspring of primates (Reitsema 2012). The offspring of *Ateles geoffroyi* began their weaning at 4 and 6 months of age. Although ILM2 ingested 100% solid food at the beginning of weaning, over the following months, as the duration and frequency of milk intake decreased, solid food consumption increased. This consumption could have been due to the novelty of the palatability of wild fruits consumed; however, milk is the energy source that infants still require, as its composition varies according to the slow development of primate offspring (Arbaiza-Bayona, et al. 2022). On the other hand, it is suggested that the early onset of weaning could have been due to the PUFAs that the mother transferred during her gestation and lactation (Fig. 1).

**Figure 1:**
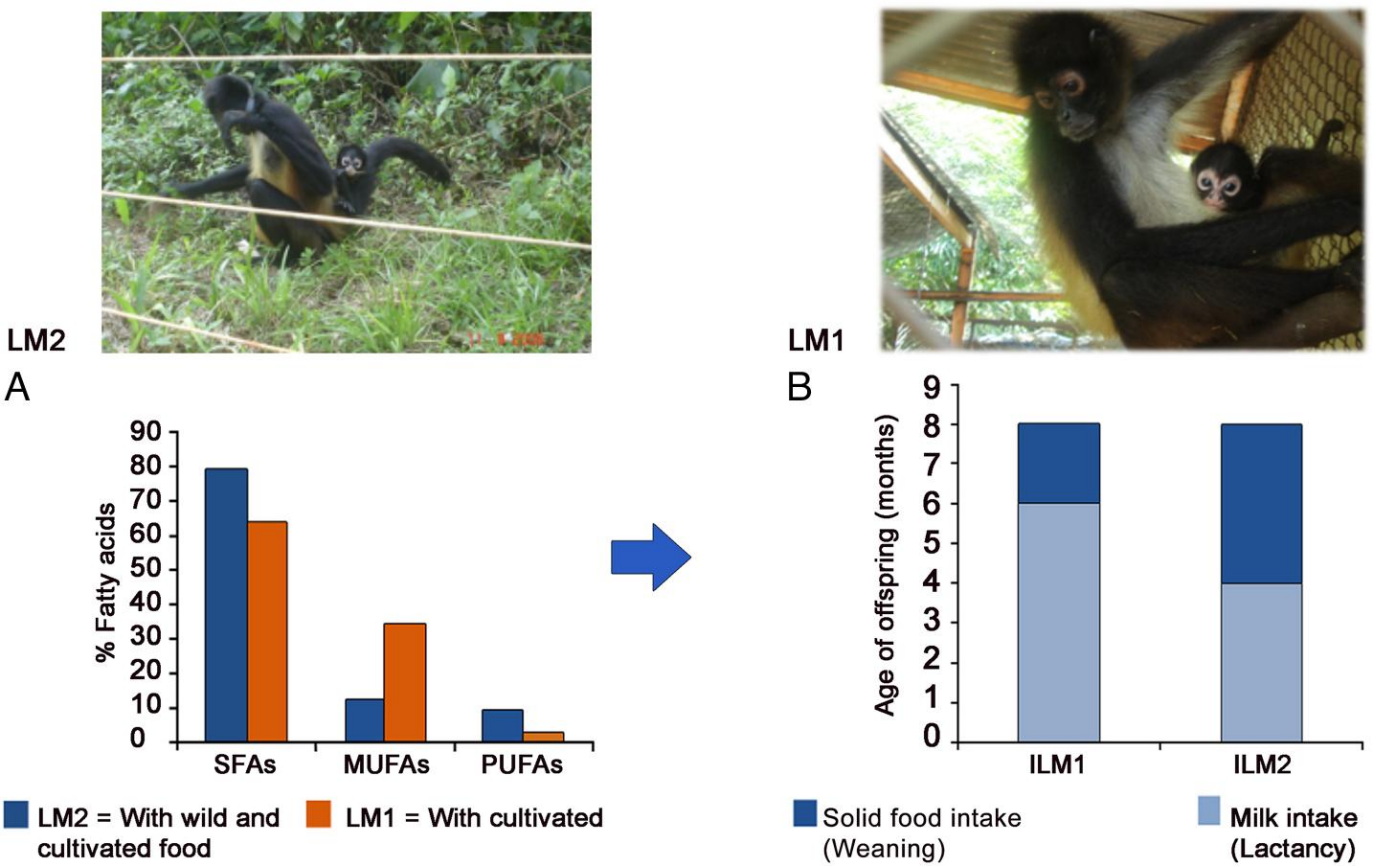
(A) Percentage of fatty acids in the milk of *A. geoffroyi* mothers: SFAs, MUFAs, PUFAs. (B) Lactation and weaning the infants ILM1, ILM2 (infant lactating mother 1, and 2). **Upper panel:**  *Ateles geoffroyi* mothers with their pups. LM1 is housed in wire-cloth cages, and LM2 is housed in an electric enclosure. **Lower panel.** The graph on the left shows the percentage of fatty acids in the milk of lactating mothers. The graph on the right shows the age of the offspring consuming milk only and the beginning of weaning (consuming milk and solid food) of these. ILM1 began weaning at 6 months of age, and ILM2 began weaning at 4 months of age.

Regarding the late onset of ILM1 weaning, it could be due to the low concentration of PUFAs in her mother's milk (Fig. 1); the diet the female consumed is rich in carbohydrates and low in lipids. Studies report that *A. geoffroyi* offspring begin locomotion between 8 and 10 months of age; before this period, they always remain nearby and in contact with their mothers. During this period, they learn to observe what the mother selects to eat (Brown et al. 2005), such as feeding on ripe fruit and the ability to discriminate between these according to dietary quality. The solid food ILM1 likely ate did not provide her with the energy she needed, so she increased her breast milk intake again. This suggests that these changes occur because of trial and error and/or inherited tendencies (Milton and Hopkins 2005).

The present results allow us to conclude that it is necessary to add seeds or some supplements rich in omega acids, which are the natural source of PUFAs and LC-PUFAs, to the diet of spider monkeys maintaining in manage cage, to improve the quality of the food provided, especially to mothers during gestation and lactation period.

## Supplementary Material

zoaf061_Supplementary_Data

## Data Availability

Data from this study are available from the corresponding author upon reasonable request.
