# Supplementary material for: Fatty acids in breast milk during lactation in Ateles geoffroyi offspring in managed care and semi-free-ranging
Source: Curr Zool. 2025 Sep 8;72(1):147–51. doi: 10.1093/cz/zoaf061 (PMC13045816; doi:10.1093/cz/zoaf061)
Supplement: zoaf061_Supplementary_Data [file zoaf061_supplementary_data.docx]

***Determination of fatty acids***

***GC-MS Analysis***

The determination of fatty acids from liquid samples of milk was carried out through gas chromatography coupled with mass spectrometry. Was used a gas chromatograph apparatus Agilent Technologies™, model 6890 N (Net Work GC system) equipped with a column DB-5, 5 % phenylmethyl polysiloxane (Agilent Technologies), 60 m long, 0.25 mm internal diameter and 0.25 μm film thickness. From each sample, 1 ml of milk was extracted v/v (volume/volume) with chloroform and ethanol in a ratio of 3:1; then the methyl esters were prepared according to the method proposed by *Egan, et al.* (*1981*), using trifluoride methanol at 14% (BF3- MeOH J.T.Baker® Xalostoc Edo. de México, Mex). We performed the extraction of methyl esters with 1.0 ml of HPLC grade hexane (C_6_H_14_ Marc® Chemical Mexico, Mex). 1 ml of hexane extract obtained from each sample at the end of the esterification, was injected into the gas chromatograph (Hewlett Packard brand®, model G1800B, GCD system; Agilent Technologies S.A., Mexico). The esters were separated on a Carbowax capillary column (Varian ®, SA, Mexico) 30 meters long, 0.25 mm internal diameter and 0.25 microns film thickness. The temperature was set at 80 °C, then raised to 250 °C at a heating rate of 30 °C/min for 25 min. Helium was used as carrier gas at a rate of 1 mL/min, and the injector temperature was 250 °C, with split injection at a ratio of 50:1. Once the chromatogram had been obtained, the identification of each peak was performed with mass spectrometry. Mass spectra were obtained through electron impact ionization at 70 eV, and compared to identify the mass spectra obtained for each compound with a database (HP Chemstation NIST-05 Mass Spectral Search program Version 2.0d). In addition, the results were compared with a standard (FAME mix, C8: C22, no. 18 920-1AMP catalog, Sigma-Aldrich ®, Mexico City) and analyzed under the same conditions, after which they were used as external standard for the quantification of fatty acids. The results are presented as total concentration and percentage.
